# Condom Failure Among HIV-Negative Men in Serodiscordant Relationships in Australia, Brazil, and Thailand

**DOI:** 10.1007/s10461-024-04431-x

**Published:** 2024-07-24

**Authors:** James Gray, Garrett Prestage, Fengyi Jin, Nittaya Phanuphak, Ruth K. Friedman, Christopher K. Fairley, David J. Templeton, Iryna Zablotska-Manos, Jennifer Hoy, Mark Bloch, David Baker, Graham Brown, Andrew Grulich, Benjamin Bavinton, for the Opposites Attract study group, Fengyi Jin, Nittaya Phanuphak, Jennifer Hoy, Mark Bloch, David Baker, Andrew E Grulich, Iryna B Zablotska, Garrett P Prestage, Benjamin R Bavinton, Beatriz Grinsztejn, David A Cooper, Anthony Kelleher, Sean Emery, Christopher K Fairley, David Wilson, Kersten K Koelsch, Kathy Triffitt, Nicolas Doong, David J Templeton, Anna McNulty, Catherine Pell, Ban Kiem Tee, Richard Moore, Norm Roth, David Orth, Angie N Pinto

**Affiliations:** 1 Kirby Institute, UNSW Sydney, Sydney, Australia; 2 Institute of HIV Research and Innovation, Bangkok, Thailand; 3 Instituto Nacional de Infectologia Evandro Chagas, Fundação Oswaldo Cruz, Rio de Janeiro, Brazil; 4 Melbourne Sexual Health Centre, Melbourne, Australia; 5 Central Clinical School, Monash University, Melbourne, Australia; 6 Department of Sexual Health Medicine and Sexual Assault Medical Service, Sydney Local Health District, Sydney, Australia; 7 Discipline of Medicine, Central Clinical School, Faculty of Medicine and Health, The University of Sydney, Sydney, Australia; 8 Westmead Clinical School, Faculty of Medicine and Health, University of Sydney, Sydney, Australia; 9 Western Sydney Sexual Health, Western Sydney Local Health District, Sydney, Australia; 10 Department of Infectious Diseases, Alfred Hospital and Monash University, Melbourne, Australia; 11 Holdsworth House, Sydney, Australia; 12 East Sydney Doctors, Sydney, Australia; 13 Centre for Social Impact, UNSW Sydney, Sydney, Australia

**Keywords:** HIV, Condoms, Gay men, Men who have sex with men, Sexual risk behaviour, Relationships

## Abstract

Condoms continue to be used by many gay, bisexual, and other men who have sex with men (GBM) to reduce the risk of HIV transmission. However this is impacted by condom failure events, defined here as condom breakage and slippage. In a prospective, observational cohort study of 343 HIV serodiscordant male couples recruited through high HIV caseload clinics and hospitals between 2012 and 2016 in Australia, Brazil, and Thailand, condom failure rates and associated factors were analysed, including with the study partner versus other sexual partners. There were 717 reported instances of condom failure from an estimated total of 25,831 sex acts with condoms, from over 588.4 participant years of follow up. Of the HIV-negative partners (n = 343) in the study, more than a third (n = 117, 36.7%) reported at least one instance of condom failure with any partner type during study follow-up. Condom failure with their study partner was reported by 91/343 (26.5%) HIV-negative partners, compared with 43/343 (12.5%) who reported condom failure with other partners. In total, there were 86 events where the HIV-negative partner experienced ano-receptive condom failure with ejaculation, representing 12.0% of all failure events. In multivariable analysis, compared to Australia, HIV-negative men in Brazil reported a higher incidence risk rate of condom failure (IRR = 1.64, 95%CI 1.01–2.68, p = 0.046) and HIV-negative men who reported anal sex with other partners reported an increased risk of condom failure compared with men who only had sex with their study partner (IRR = 1.89, 95%CI 1.08–3.33, p = 0.025). Although at least one event of condom failure was reported by a significant proportion of participants, overall condom failure events represented a small proportion of the total condom protected sex acts.

## Introduction

Condoms are an important strategy in supporting good sexual and reproductive health, with gay bisexual, and other men who have sex with men (GBM) using them for the prevention of sexually transmissible infections (STIs), and for HIV. Maintaining high levels of condom use remains a significant priority in the global HIV prevention strategy for UNAIDS [1]. However, the dynamics of condom use for the prevention of HIV are changing due to the proven efficacy of HIV treatment as prevention (TasP) [2–4] and pre-exposure prophylaxis (PrEP) [5]. The centrality of condom use as a method to reduce HIV transmission risk among GBM is diminishing and it has become just one option alongside TasP and PrEP, albeit with the additional benefit of reducing the transmission of other STIs [6, 7].

Consistent condom use is estimated to reduce HIV transmission by 91% in GBM [8]. While generally highly effective, a global review on condom errors in both opposite- and same-sex intercourse [9] reported that condom use can be associated with errors and problems that impact on their effectiveness in real-world use. Errors include not using them during the entirety of intercourse, applying the condom incorrectly, not using water-based lubricants, and incorrect withdrawal following sexual intercourse. Condom use problems include breakage, slippage, leakage, and difficulties with maintaining an erection. This same review reported a high variation in reported condom breakage rates, from as low as 1.1% to as high as 32.8% of acts. Estimates of slippage are also highly variable with reports of per participant slippage between 13.1% and 19.3% and per condom act between 0.0% and 6.6%.

Many studies have focussed on condom use errors and problems in heterosexual men, often recruited through sexual health clinics or universities. These studies report relatively high levels of breakage (15.0–27.6%) and slippage (6.6–13.6%) in the previous three months [10, 11]. Breakage and slippage have been found to be less common in regular partners (5.1%), compared to other partners (9.4%) [12]. One study of young heterosexual men attending an STI clinic found 31.3% reported at least one condom breakage in their last three penile-vaginal sexual acts [13], and correlates of breakage were having a previous STI, condom slippage, and low self-efficacy for condom use. Breakage was also associated with allowing condoms to come into contact with sharp edges, fit or feel problems, and not squeezing air from the tip. A large cross-sectional study of GBM reported condom failure in 4.0% of episodes of anal intercourse and breakage was positively associated with younger age, a higher number of partners, and alcohol or drug use with casual partners [14]. A study of gay men attending an STI clinic reported a condom failure in 0.7% of sexual acts with main partners and 6.4% with non-main partners [15].

Compared with heterosexual people, open relationships, and HIV serodiscordant relationships are more common for GBM. Although consistent condom use is declining for GBM with both regular and other partners, it remains an important prevention tool [16–19]. The existing literature on condom failure, defined as breakage or slippage has limitations. Most of the studies come from the United States or are focused on heterosexuals in Africa, often recruited via sexual health clinics or universities, are cross-sectional, and only examine condom failure with one partner type. There are limited recent data examining condom failure in male HIV serodiscordant couples. Among HIV-negative men in serodiscordant relationships, we aimed to look longitudinally at condom failure rates with the enrolled HIV-positive study partner and other sexual partners, and identify associated factors in Australia, Brazil, and Thailand.

## Methods

### Participants

Participants were GBM in the Opposites Attract study, for which the design and methods have previously been published [20]. Briefly, data were collected as part of a prospective, observational cohort study of HIV serodiscordant male couples recruited through high caseload HIV clinics, sexual health clinics and hospitals in Australia, Brazil, and Thailand. The primary goal of the study was to determine the impact of antiretroviral therapy (ART) on the prevention of HIV transmission among GBM [2].

In order to be eligible to participate, both men in a couple, defined here as ‘study partners’, had to be at least 18 years old, one partner be HIV-positive and the other partner HIV-negative at baseline, be having anal sex at least once a month on average, and agree to attend clinic visits at least twice a year. Enrolment occurred between 2012 and 2014, with follow-up through to the end of 2016. Couples were followed up until the end of the study (n = 230) or until they became ineligible (n = 72), or they withdrew or were lost to follow-up (n = 41). Among those that became ineligible, 60 (83%) ceased within-couple anal intercourse entirely or broke up, ten (14%) reported anal intercourse less than once per month on average, and two (3%) died in separate couples.

### Procedures

Couples were required to complete online computer-assisted self-interview questionnaires which collected behavioural and attitudinal information and were aligned to the time of each study visit. The questionnaires for the two partners differed, with the HIV-negative partner asked detailed questions about their sexual behaviour. These questions were not asked of the HIV-positive partners due the potential legal impacts of public health legislation in place at the time of the study in Australia. The questionnaires were available in English, Brazilian Portuguese, and Thai. Clinical data were collected via electronic case report forms. Ethics approvals were obtained in all three countries [20].

### Measures

Demographic characteristics including age, education and ethnicity were collected from both partners, as well as information about the length of time since they first had sex with each other and whether they lived together. The HIV-negative partner was asked about their clear, spoken agreements with their study partner about sex with other men, including what this involved (may not have sex at all, may not have anal sex, may only have sex with condoms, may have sex without condoms, other). The HIV-negative partner was asked about sexual behaviour with others in the previous 3 months, whether they themselves had anal intercourse with other men, and how many times they had anal intercourse with other partners (never, once, twice, 3–5 times, 6–10 times, 11–30 times, 31–50 times, over 50 times) for both insertive and receptive anal intercourse. They were also asked how many times they had anal intercourse using a condom with different partner types including study partner, other HIV-negative partner, other HIV-positive partner, and other HIV status-unknown partner (never, once, twice, 3–5 times, 6–10 times, 11–30 times, 31–50 times, over 50 times).

HIV-negative participants were asked about condom breakage and slippage as a single option, which was termed ‘condom failure’ for the purpose of this analysis. This was followed by two questions about condom failure events in the context of insertive and receptive anal intercourse, separately; the question for each context was ‘How many times…’ (never, once, twice, 3–5 times, 6–10 times, 11–30 times, 31–50 times, over 50 times). An additional question was asked about ejaculation in the context of condom failure using the same question and response structure. These questions were asked for sex with the study partner and for sex with other partners, by perceived HIV viral load status of the partners (undetectable, low, moderate, high, he has not received the results yet, I don’t know). Several key variables were constructed from the items above. The count of condom acts and failure events were estimated by taking the midpoint of the categories. A variable of the number of couples that had any type of condom failure with different partner types at any point of follow-up was also created.

### Analysis

Data were analysed using Stata 15.1 (Stata Corporation, College Station, Texas, USA). Couples were excluded if they had no follow-up visits (n = 13). Couples that later became ineligible were included in the analysis if follow-up data was available. Descriptive analyses of condom failure variables were conducted, by partner type (study partner versus other partner), sexual position (insertive versus receptive), and HIV status of other partners. Responses from the baseline questionnaire were included in the analysis. Country differences were also explored. The mean number of sex acts per participant per country were calculated by dividing the number of reported acts of anal intercourse by the number of participants from that country. Incidence rates (IR) of condom failure for partnership types and by sexual position were calculated by dividing the number of condom failure events by the couple-years of follow-up. Where the estimate included a fraction, this was rounded up to the nearest whole number. Bivariable and multivariable generalised linear models were used to examine associations over follow-up between a range of variables and condom failure. The strengths of the associations were presented as incidence rate ratios (IRR) along with their corresponding 95% confidence intervals (95%CI), and p-values.

## Results

There were 343 HIV-negative partners who attended one or more follow-up visits and were included in the analyses totaling over 588.4 participant years of follow up. Participants were recruited from Australia (n = 153), Brazil (n = 93) and Thailand (n = 97), had a mean age of 34.1, were most likely to be university educated (55.0%), and identify as gay (92.4%). Across all partner types and across follow up, 51,983 acts of anal intercourse were reported, of which an estimated 25,831 (49.7%) were with a condom. The HIV-negative partners reported they were the insertive partner 15,586 times (60.3%) and the receptive partner for the remaining 10,245 times (39.7%). Brazilian HIV-negative partners reported the highest mean number of sex acts with condoms over the entire study follow-up (n = 9539, mean = 102.6, standard deviation [SD] = 14.3), followed by Thai men (n = 6840, mean = 70.5, SD = 13.4) and Australian men (n = 9452, mean = 61.8, SD = 14.4).

**Fig. 1:**
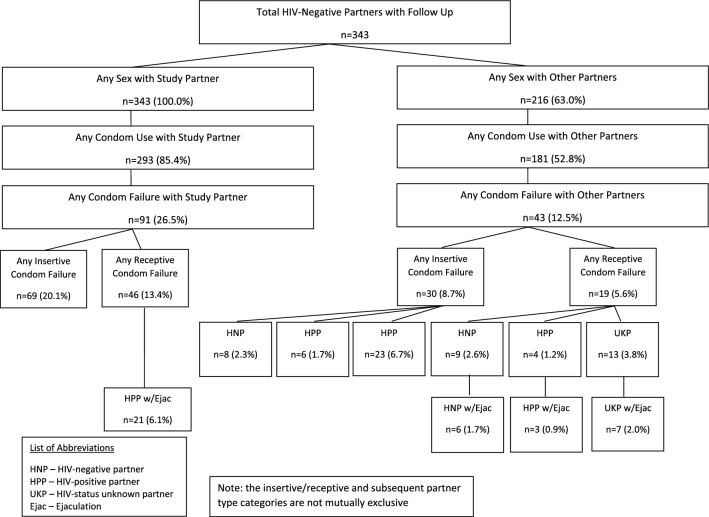
Condom failure reported by HIV-negative partner over follow up

As outlined in Fig. 1, 293 (85.4%) HIV-negative partners reported any condom use with their study partner across follow-up. Ninety-one (26.5%) reported any condom failure with their study partner, with 69 (20.1%) reporting condom failure during insertive intercourse and 46 (13.4%) reported condom failure during receptive intercourse. Twenty-one HIV-negative partners (6.1%) reported receptive condom failure with ejaculation at least once. Any sex with other partners was reported by 216 (63.0%) HIV-negative partners across follow up and 181 (52.8%) reported any condom use with other partners. Forty-three (12.5%) reported any condom failure with other partners over follow-up. Thirty (8.7%) HIV-negative partners reported condom failure during insertive intercourse, with eight (2.3%) with HIV negative partners, six (1.7%) with HIV-positive partners, and 23 (6.7%) with partners of unknown HIV status. Nineteen (5.6%) HIV-negative partners reported receptive condom failure. Nine (2.6%) reported condom failure over follow up a known HIV-negative partner and ejaculation was reported to have occurred at least once by six men (1.7%). Four (1.2%) reported condom failure over follow up with a known HIV-positive partner and ejaculation was reported by three (0.9%). Finally, there 13 (3.8%) reported condom failure with a partner of unknown HIV status and ejaculation was reported by seven (2.0%). At least one condom failure event with either their study partner or other partners was reported by 117 (36.7%) participants.

Condoms failure events were relatively rare within each three month period of follow up. They were reported only once (n = 99), or twice (n = 60), three to five times (n = 35), six to ten times (n = 9), 11–30 times (n = 7), 31–50 time (n = 3), and over 50 times (n = 1). Over a total of 408.6 person-years under follow-up where condoms were used, the HIV-negative partners reported an estimated 717 condom failure events with any partner type across follow-up (Table 1), with an IR of 1.75 (95%CI: 1.63–1.89) condom failure events per person-year of follow-up, split between an estimated 468 insertive condom failure events (IR = 1.37, 95% CI 1.23–1.51), and an estimated 249 receptive condom failure events (IR = 0.95, 95% CI 0.83–1.07). Condom failure events when in the insertive position were more common with other partners (n = 184, IR = 1.35, 95% CI 1.16–1.56) compared to study partners (n = 285, IR = 1.02, 95% CI 0.90–1.14). Insertive condom failure events varied between Australia (n = 101, IR = 0.71, 95% CI 0.58–0.86), Brazil (n = 161, IR = 1.55, 95% CI 1.32–1.81), and Thailand (n = 206, IR = 2.20, 95% CI 1.91–2.52). Condom failure events when in the receptive position were less common with other partners (n = 64, IR = 0.61, 95% CI 0.47–0.79) compared to study partners (n = 186, IR = 0.89, 95% CI 0.77–1.03). Receptive condom failure events varied between Australia (n = 72, IR = 0.62, 95% CI 0.48–0.78), Brazil (n = 91, IR = 1.08, 95% CI 0.87–1.32), and Thailand (n = 83, IR = 1.41, 95% CI 1.14–1.77). In total, there were 86 events of receptive condom failure with ejaculation, representing 12.0% of all condom failure events, and 34.5% of all receptive condom failures. Of these, 39 (45.3%) were with their HIV-positive study partner, seven (8.1%) were with other known HIV-positive partners, 22 (25.6%) were with HIV status unknown partners and the remaining 18 (20.9%) were with HIV-negative partners.

**Table 1:** Incidence rates of condom failure reported by HIV-negative partner

	Estimated number of sex acts with condoms across follow up	Estimated number of condom failure events	Condom failure rate per 100 sex acts	Person-years under follow up	Condom failure incidence Rate (IR) per person year
All partners	26,061	718	2.87	408.64	1.75 (1.63–1.89)
Insertive	15,755	469	2.97	341.30	1.37 (1.23–1.51)
Receptive	10,306	249	2.42	262.72	0.95 (0.83–1.07)
Study partner	19,707	471	2.39	350.87	1.34 (1.22–1.47)
Insertive	11,984	285	2.38	280.97	1.02 (0.90–1.14)
Receptive	7723	186	2.40	207.72	0.89 (0.77–1.03)
Other partners	6355	248	3.89	164.02	1.51 (1.33–1.71)
Insertive	3772	184	4.87	136.27	1.35 (1.16–1.56)
Receptive	2583	64	2.46	103.70	0.61 (0.47–0.79)
By Country (all partners)					
Australia	9488	173	1.82	179.03	0.97 (0.83–1.12)
Insertive	5353	101	1.89	142.99	0.71 (0.58–0.86)
Receptive	4135	72	1.74	116.55	0.62 (0.48–0.78)
Brazil	9611	256	2.66	121.56	2.10 (1.85–2.37)
Insertive	5582	161	2.88	104.27	1.55 (1.32–1.81)
Receptive	4029	95	2.35	87.72	1.08 (0.87–1.32)
Thailand	6964	290	4.15	108.04	2.68 (2.39–3.01)
Insertive	4821	207	4.28	94.04	2.20 (1.91–2.52)
Receptive	2143	83	3.85	58.45	1.41 (1.14–1.77)

There were multiple bivariable associations with condom failure events (Table 2). Briefly, by country, compared to Australia, HIV-negative men in Brazil (IRR = 2.39, 95% CI 1.21–4.71, p = 0.012) and Thailand (IRR = 3.25, 95% CI 1.42–7.44, p = 0.005) reported significantly higher incidence of condom failure. More condom failure events were reported by: HIV-negative men who first had sex with their study partner between one and five years prior to the study (IRR = 3.00, 95% CI 1.19–7.54, p = 0.020), compared to those who first sex had been less than a year previously, or more than 5 years previously; men who had recently condomless anal intercourse (CLAI) with their study partner (IRR = 2.94, 95% CI 1.78–4.85, p < 0.001) compared to those that consistently used condoms; men who had anal sex with other partners (IRR = 2.51, 95% CI 1.43–4.43, p = 0.001) compared to those that were monogamous; and men who reported PrEP use in the previous three months (IRR = 1.78, 95%CI:1.00–3.15, p = 0.049). HIV-negative men aged over 40 were significantly less likely to report condom failure (IRR = 0.40, 95% CI 0.17–0.91, p = 0.029). In multivariable analysis, by country, compared to Australian men, Brazilian men had increased likelihood of condom failure (aIRR = 1.64, 95% CI 1.01–2.68, p = 0.045), while the association was no longer significant for men in Thailand (aIRR = 3.47, 95% CI 0.96–12.57, p = 0.058). Men who had anal sex with other partners had greater risk of condom failure events (aIRR = 1.89, 95% CI 1.08–3.33, p = 0.025) compared to those that were monogamous.

**Table 2:** Bivariable and multivariable generalised linear model analysis of predictors of any condom failure with any partner

Variable	Any condom failure—bivariable (IRR, 95%CI)	p-value	Any condom failure—multivariable (aIRR, 95%CI)	p-value
Country				
Australia	1		1	
Brazil	**2.39 (1.21–4.71)**	**0.012**	**1.64 (1.01–2.68)**	**0.046**
Thailand	**3.25 (1.42–7.44)**	**0.005**	3.47 (0.96–12.57)	0.058
Age of HIV-negative partner				
Under 30 years	1		1	
30–39 years	0.81 (0.34–1.91)	0.624	0.90 (0.34–2.68)	0.834
40 years and over	**0.40 (0.17–0.91)**	**0.029**	0.65 (0.25–1.68)	0.375
Education of HIV-negative partner				
High School or less	1		1	
Vocational	0.36 (0.12–1.04)	0.058	0.84 (0.43–1.67)	0.637
University	0.56 (0.17–1.83)	0.339	1.20 (0.49–2.93)	0.687
HIV-negative partner employed full-time				
No	1	0.914	1	0.487
Yes	0.94 (0.33–2.73)		0.67 (0.21–2.09)	
Study partners live together full-time				
No	1		1	0.117
Yes	0.79 (0.46–1.33)	0.373	0.48 (0.15–1.24)	
First sex within the couple				
Less than 12 months	1		1	
1–5 years	**3.00 (1.19–7.54)**	**0.020**	2.01 (0.45–8.89)	0.385
5 or more years	0.79 (0.39–1.58)	0.502	1.80 (0.70–4.67)	0.225
Agreement to have CLAI inside relationship				
No	1	0.065	1	0.378
Yes	2.20 (0.95–5.11)		2.47 (0.33–18.45)	
Having CLAI with study partner				
No	1	**< 0.001**	1	0.227
Yes	**2.94 (1.78–4.85)**		1.39 (0.81–2.37)	
Perceived study partner to have detectable VL				
No	1	0.329	1	0.756
Yes	1.44 (0.69–2.98)		1.10 (0.461–1.97)	
Anal Sex with other partners				
No	1	**0.001**	1	
Yes	**2.51 (1.43–4.43)**		**1.89 (1.08–3.33)**	**0.025**
PrEP use in previous 3 months				
No	1	**0.049**	1	0.749
Yes	**1.78 (1.00–3.15)**		1.23 (0.33–4.48)	

Bolded results indicate *p* < 0.05

## Discussion

Condom failure was relatively uncommon in this cohort, accounting for less than 3% of sex acts where condom use occurred. Condom failure was more common with other partners than study partners, including a higher proportion of receptive condom failure with ejaculation with other partners and it was more common in Brazil and Thailand than in Australia.

Within the existing literature the proportion of gay men reporting condom failure varies significantly and rarely differentiates between regular and casual partners. Among several studies conducted in the 1990s, 13.4% [21] of respondents had experienced condom failure at least once in the previous year, found that 16.6% [22] reported condom failure in the previous six months, and a failure rate of 2.1 per 100 condom protected acts, and 38% reported condom failure in an 18 month period. More recent surveys of GBM have found similarly diverse experiences of condoms failure ranging from 4% [14] to 31% [23]. When looking at the condom failure rates for other populations, the range is similarly large [9]. The results of this study regarding condom failure fall within the large ranges previously reported in the literature for GBM, however, demonstrates this specifically for HIV-serodiscordant male couples with both regular and casual sex partners. While a higher proportion of HIV-negative partners reported any condom failure with their study partner than with other partners, the incidence rate of condom failure was higher with other partners, which aligns with the existing literature [12, 15].

Close to half of sex acts in this study occurred with condom use. In our study, the HIV-negative partner reported more condom-protected insertive sex acts compared with receptive acts. The proportion of HIV-negative men reporting condom failure was higher for insertive sex rather than receptive sex, and this was consistent between partner types. There are a number of factors associated with condom failure [9, 13, 24], which may be more typically associated with the actions of the insertive partner if they are taking primary responsibility for applying the condom. Potentially, these men were less cautious when applying the condom. Alternatively, it is likely that condom failure is primarily noticed by the man in the insertive role; if the person in the receptive role is not told, they may not be aware of what has happened, and this may also lead to a reporting bias. Conversely, they may have been more conscious of problems applying and using the condom when they were at potentially increased risk of HIV acquisition in the receptive anal intercourse role. In the case of the HIV-positive study partners, they may have been more motivated to protect their partner [25, 26] and taken greater care in their use. Connected to this, HIV-negative men had a higher incidence of failure when having sex with other partners in comparison to their study partner and this corresponded with having a higher rate of condom failure risk in the multivariable analysis. This may be a result of less familiarity between the partners leading to actions that present a greater risk of failure.

Although there were relatively few condom failure events that occurred with ejaculation (n = 86), they were a high proportion of the receptive failure events. Of the receptive failure events with their study partner, 45.7% occurred with ejaculation. Numbers were small with other partners, however 66.7% of failures with other HIV-negative partners included ejaculation, 75.0% with other HIV-positive partners and 53.8% of unknown partners. The high level of treatment and undetectable viral load among the HIV-positive study partners, mean that despite the higher rates of condom failure within the relationship, the HIV transmission risk was very low (although this had not been definitively proven for GBM at the time). There was risk from outside partners; as has previously been published, the only seroconversions that occurred in this study were from outside partners [2]. It is not possible to link those transmissions to condom failure, but this illustrates that risk of HIV acquisition for HIV-negative partners in HIV serodiscordant male couples has shifted to outside the relationship [2, 27]. Additionally, information about the impact of HIV treatment on transmission was published during data collection which likely had an impact on participant perceptions of risk [2, 3], and there was an increase in viral load agreements within the study couple and less condom use [27]. Further, over follow-up there was a move towards agreements that allowed sex outside the relationship and for less condom use [28].

Although the incidence risk rate for condom failure was lower for sex with the study partner compared to other partners, a higher proportion of HIV-negative partners reported condom failure with their study partner, which could relate to a number of factors. The likely higher level of trust could make people less focused on correct condom usage. There could also be an impact from repeated condom failures which had not led to seroconversion, which in the context of undetectable viral load would give reassurance that HIV acquisition is unlikely.

Condom type, size and fit could also play a role in these findings. For instance, condoms that are too large are associated with slippage and condoms that are too small are associated with difficulties in maintaining an erection which may lead to partial usage [9], and availability may vary by country.

HIV-negative men had a higher incidence risk rate for condom failure in Brazil and Thailand and this may reflect differences in access and education regarding condom use, particularly for GBM. In Thailand, concerns have been raised about the quality and consistency of sexual health education, including regarding condom use [29]. Consistent lubricant use for anal sex among Thai men who have sex with men has been reported to be 77%, with consistent use negatively associated with inconsistent condom use, binge drinking, and purchasing sex [30]. Additionally, age may have also played a factor as the couples in Brazil and Thailand were younger than those in Australia [2].

Beyond ensuring high quality condoms are being produced and distributed, in the context of the HIV-negative partners who experience repeated condom failures, there are two primary interventions that could reduce risk: (1) improved condom education and support to address the risk factors for condom failure; and (2) utilising PrEP (which would depend on the willingness of the individual to use it). These two interventions could be offered as options but are also dependent on the individual seeing condom failure as of sufficient risk to prompt action and engagement with either health or community-based services to seek assistance.

Studies of condom effectiveness for sexually transmissible infections in community settings can have a large range of limitations [31]. The definition of consistent condom use can be varied [32] and typically relies on self-report which can be subject to reporting bias [33–35]. In our study, condom use, and condom failure were self-reported, which comes with recall bias potentially leading to differential over-reporting of failure, although the regularity of data collection would have mitigated this to some degree. It also comes with social desirability bias, although this would have been somewhat mitigated by the participants being informed that their clinicians would never see their individual data. Additionally, this analysis classified participants by any reported condom usage with study and other partners, and future analyses should investigate whether condom failure was related to the regularity of condom use by these men. The sample is biased towards couples that were willing to participate in a multiyear study with significant time costs. Detail about the type of sex with outside partners was limited to the HIV-negative partner, while for HIV-positive partners we only knew if they have had sex with other partners. However, as the focus of this paper is the HIV risk for HIV-negative partners, this gap is less relevant.

## Conclusion

Condom failure presents an ongoing risk for HIV-negative men in serodiscordant relationships where the HIV-positive partner is not virally supressed, or the HIV-negative partner is utilising condoms as their main prevention strategy with other sexual partners. The higher incidence of condom failure events in insertive anal sex with the study partner and with outside partners, also observed across countries, likely reflects the lower HIV risk perception attributed to this sexual practice. For the small group of men experiencing larger numbers of condom failure events, education on correct condom use remains important. Alternatively, HIV PrEP could be discussed as an option and strongly considered.

## Data Availability

A Research Data Management Plan has been developed for this research. All data is held within a university data repository to ensure transparency.

## Code availability

Analysis was undertaken via StataIC 15 (64-bit).
